# Well-being as a primary endpoint in clinical trials: a call for consensus

**DOI:** 10.1038/s44184-026-00234-1

**Published:** 2026-08-06

**Authors:** Elliot Marseille, Hannes Kettner, Tyrone J. Sgambati, James G. Kahn, Stefano Bertozzi, Will Lucas, Robin Carhart Harris, Dacher Keltner, Charles Raison

**Affiliations:** 1 Collaboration for the Economics of Psychedelics, University of California Berkeley, Berkeley, CA USA; 2 Imperial College, London, UK; 3 UC Berkeley, Center for the Science of Psychedelics, University of California, Berkeley, Berkeley, USA; 4 Global Mental Health, University of California San Francisco, San Francisco, CA USA; 5 School of Public Health, University of California Berkeley, Berkeley, CA USA; 6 Carhart-Harris Lab, University of California San Francisco, San Francisco, CA USA; 7 Department of Psychiatry, University of Wisconsin-Madison, Madison, WI USA

**Keywords:** Diseases, Health care, Medical research, Psychology, Psychology

## Abstract

Psychiatric trials typically define success as symptom reduction, yet patients prioritize meaning, vitality, and functioning. This imbalance undervalues experiential benefits, distorting clinical, policy, and FDA decisions. Psychedelic trials illustrate this, where robust well-being gains sometimes accompany modest symptom effects. We argue well-being should be elevated to co-primary endpoint status, propose a Delphi-developed consensus well-being instrument with rigorous validation, and outline guardrails preventing approval of therapies that worsen the underlying condition.

## Background

The World Health Organization defines health as “a state of complete physical, mental and social well-being and not merely the absence of disease or infirmity”^1^. This argument extends a long-standing salutogenic orientation in health promotion: the view, originating with Antonovsky, that health is not merely the absence of disease but the presence of well-being and of resources to cope with life’s demands^2^. A recent JAMA Viewpoint has called for an explicitly salutogenic focus in psychiatry^3^, a reorientation that our proposal advances into the domain of regulatory measurement science. Yet, in clinical trials, especially in mental health, primary endpoints typically focus on symptom reduction, treating the absence of illness as the ultimate goal. Measures of well-being, such as work and social functioning and positive mental states (e.g., life satisfaction, optimism, a sense of connectedness, and overall well-being), are often relegated to secondary or exploratory endpoints, if measured at all. A 2015 systematic review of psychotropic medication outcomes found that most studies focused on symptoms and side-effects, with scant attention to functioning or broader life impacts. Outcomes were rarely elicited from patients, nor did studies adopt a patient perspective^4^. A 2020 international online survey of 3003 patients, informal caregivers, and clinicians with experience of depression identified 80 outcome domains related to treatment benefit and 57 more relating to safety, care organization, and stigma. Many of the frequently cited domains (e.g., mental pain, social isolation, capacity to work, and family life) are not covered by the most common depression scales, including HAM-D, Montgomery–Åsberg Depression Rating Scale (MADRS), Beck Depression Inventory (BDI), Inventory of Depressive Symptomatology/Quick Inventory of Depressive Symptomatology (IDS/QIDS), *CES-D*—Center for Epidemiologic Studies Depression Scale (CES-D), and Patient Health Questionnaire-9 (PHQ-9)^5^. Participants described depression remission as an opportunity for personal growth and restoration of social and vocational roles, not merely a return to baseline symptoms, outcomes closer to enhanced well-being than to symptom absence.

The FDA increasingly emphasizes clinical assessments, including patient-reported outcomes, that reflect how patients feel and function in daily life^6,7^. In psychiatry, many commonly used symptom scales already function as patient-reported outcomes. However, these instruments are designed primarily to quantify reductions in negative symptoms, such as depressed mood, anxiety, or intrusive thoughts, rather than gains in positive mental health. This asymmetry underscores the need for a validated pathway through which positively valenced well-being measures can assume primary or co-primary endpoint status.

Multiple studies have found that diverse well-being constructs—from life satisfaction to positive affect and psychological quality of life—largely represent a single underlying factor, sometimes called “general well-being”^8,9^. This suggests that “well-being” can be quantified as a unifying metric, simplifying its adoption as a primary endpoint. Recent NIH-supported work on “emotional well-being” reaches similar conclusions, proposing an umbrella construct that integrates experiential (momentary affective) and reflective (life satisfaction, meaning, goal pursuit) components of psychological well-being under a single multi-dimensional framework^10^. Thus, we use the term “well-being” to refer to a general factor that integrates positive affect, life satisfaction, and psychological functioning. This changing landscape sets the stage for a critical policy question: *Should measures of well-being be elevated to primary endpoints in clinical trials and FDA applications?* Nowhere is this question more salient than in the emerging field of psychedelic therapy.

Psychedelic trials (e.g. with psilocybin and 3,4-methylenedioxymethamphetamine (MDMA)) often show not only symptom improvement but also gains in meaning, social connectedness, and functioning, including health-related behavior change^11^. However, in FDA-oriented trial reporting these effects are typically treated as secondary, rather than central indicators of benefit. We focus on psychedelic-assisted therapies not because they require well-being endpoints to demonstrate efficacy; several trials have shown robust effects on conventional symptom measures, including, psilocybin for depression^12–15^, the indication with the largest evidence base, as well as MDMA-assisted therapy for Post-Traumatic Stress Disorder (PTSD)^16^ and psilocybin for alcohol^17^ and tobacco use disorders^18^. Rather, psychedelic-assisted therapies illustrate a broader problem: even when symptom endpoints succeed, they do not capture the full scope of patient-prioritized outcomes. This concern generalizes beyond psychedelics: any intervention with experiential and functional benefits will be incompletely characterized by symptom reduction alone. Symptom reduction alone is also blind to timing: beneficial changes in functioning, connectedness, and meaning may precede symptom change, particularly in the time-limited window of a clinical trial (see “Well-being as both an endpoint and a pathway,” below).

We argue that it is time to formally elevate validated well-being measures to primary outcome status in clinical trials, especially in mental health. Doing so would align trial evidence with the full spectrum of patient-valued outcomes and could have far-reaching implications for regulatory approval, health technology assessment, reimbursement, and access to novel therapies.

## Problem statement

Given the mismatch between trial endpoints and patient priorities, several clinical and policy consequences follow:

### Value for prognosis

Well-being appears to have prognostic value. Longitudinal studies show that higher well-being predicts lower risk of subsequent depression^19–22^, higher rates of recovery after episodes^23,24^, reduced suicidality^25,26^, and, in some cohorts, fewer medical visits^27,28^, often even after adjusting for baseline symptom severity. In this sense, elevating well-being as a primary endpoint is analogous to treating LDL cholesterol as a primary endpoint for statins. Clinicians do not prescribe statins merely because “low LDL is good,” but because lowering LDL predicts fewer heart attacks and deaths. Similarly, demonstrating gains in well-being matters because those gains are plausibly linked to better long-term mental health and functioning.

### Undervaluing benefit

When well-being and related outcomes are treated as secondary, trials may underestimate the benefits of an intervention. A treatment might enhance a patient’s quality of life, sense of purpose, or ability to thrive, but the primary symptomatic outcome won’t reflect these gains. This pattern is not unique to psychiatric trials: across medicine, prominent RCTs have “failed” on hard clinical primary endpoints yet demonstrated meaningful improvements in secondary patient-reported symptoms, function, or quality of life. Among these are trials of vagus nerve stimulation^29,30^, ischemia^31,32^, obstructive sleep apnea^33^, atrial fibrillation^34^, and stable coronary disease^35^. Beyond simple undercounting, this measurement asymmetry can distort the evidence base. Two treatments may appear equivalent because they produce similar reductions on a symptom scale, even if one leaves patients functionally diminished while the other restores high life satisfaction and engagement^36^.

When well-being outcomes are designated as secondary, they are often not incorporated into sample-size calculations and may be tested with multiple-comparison adjustment (i.e., at a smaller effective alpha). Thus, even clinically meaningful improvements can fail to reach significance thresholds. This is largely a design artifact rather than evidence of limited therapeutic value. Pre-specifying well-being as a co-primary endpoint, or as a hierarchically tested key secondary endpoint, would force upfront planning of multiplicity and sample size so trials are adequately powered to detect patient-salient change.

### Regulatory impact

Regulators, such as the FDA, base approval and labeling primarily on primary endpoints. If well-being is not a primary outcome, improvements in positive mental health may not make it into the drug’s label or the core evidence used for approval. This means a therapy that improves patients’ lived experience might be judged by regulators as having marginal benefit if it only modestly changes a symptom scale. FDA’s Patient-Focused Drug Development initiative and the 21st Century Cures Act have formalized expectations that sponsors measure outcomes reflecting how patients feel and function—and many commonly used psychiatric symptom measures are clinical outcome assessments, including patient-reported outcomes (PROs)^37,38^. However, these frameworks have been applied primarily to illness-anchored constructs: symptom burden, functional impairment, treatment tolerability. Whether positively-valenced well-being—measuring flourishing rather than reduced suffering—can serve as primary evidence of benefit for a disease indication remains untested. The evidentiary pathway exists; no sponsor has yet navigated it. (See Supplementary Material for detailed regulatory context.)

### Health economic consequences

Economic evaluations typically summarize health gains as Quality-Adjusted Life-Years (QALY) derived from health-utility instruments (e.g. time trade-off, EQ-5D). These are not designed to capture the full range of positively-valenced mental well-being and generally impose an upper bound at “full health.” Gains in meaning, social connectedness, vitality, and life satisfaction may be missed or only weakly reflected in utilities, biasing cost-effectiveness results against interventions whose principal benefits are experiential and functional. This has real-world resource implications: higher happiness and affective well-being tend to coincide with better work performance, and higher life satisfaction predicts fewer medical visits^27,39^, suggesting that well-being changes may also be relevant to productivity and utilization components of economic evaluation. Thus, current QALY practice may undercount patient-prioritized well-being gains and, in turn, understate both health and economic benefits (including productivity and downstream utilization).

A parallel Health Technology Assessment (HTA)-oriented effort is the EuroQol Health and Wellbeing instrument (EQ-HWB), a new preference-based instrument intended to generate QALYs while covering broader well-being domains (e.g., relationships, autonomy) than EQ-5D^40^. EQ-HWB remains anchored to the conventional 0-1 utility scale and therefore likely underestimates the absolute gain in benefits over a traditional symptom-based approach.

### Measurement drives focus and investment

Outcomes specified and measured by policy and programs encourage an increase in those specified outcomes^41–43^. In antidepressant development, regulators and sponsors have historically judged success almost entirely by reductions in depression scores and relapse rates, with little attention to domains such as intimacy, sexual functioning, or relationship quality. Yet the latter domains matter enormously to patients and partners, and they are likely to influence long-term adherence and overall life satisfaction. For example, qualitative work in depression care finds that patients and clinicians converge on outcomes such as social functioning, quality of life, and achievement of personal goals as central indicators of successful treatment, whereas conventional trial endpoints focus largely on symptom scales and remission/response status^44,45^.

Meta-analytic data in pediatric depression show that while antidepressants produce small gains in functioning, they show no clear benefit for Quality of Life (QoL), and QoL is rarely measured at all^46^. Importantly, “QoL” in antidepressant trials is typically operationalized as *health-related* QoL: composite measures dominated by symptom burden and functional limitations (e.g., problems in daily roles, work/social participation), where the best possible score often corresponds to “no problems” rather than positive well-being. As a result, these measures are relatively insensitive to positively valenced outcomes—such as vitality, connectedness, meaning, optimism, and personal growth—that patients often describe as central to recovery. Another recent meta-analysis of 46 double-blind placebo-controlled antidepressant RCTs found only small effects on quality of life (Standard Mean Difference ≈ 0.22) and, critically, a strong correlation between QoL effects and depression symptom effects (*ρ* ≈ 0.73). The authors conclude that ‘… the current practice of measuring QoL may not provide sufficient additional insights into the well-being of patients’ beyond symptomatic recovery. In other words, the way QoL is currently operationalized in antidepressant trials adds little beyond symptom scales—exactly the limitation we aim to address by developing and validating a distinct, positively-valenced well-being measure^47^.

## Evidence from psychedelic trials

Psychedelic science provides a compelling case study for why positive well-being measures should be primary endpoints. Trials of psychedelic-assisted therapy (e.g., for depression, PTSD, end-of-life anxiety) have consistently reported patient-reported benefits that extend well beyond symptom relief. However, these benefits often hide in secondary outcomes or qualitative reports^48^. Their exclusion from primary analyses likely underrepresents the total therapeutic effect associated with these treatments.

### MDMA for PTSD and anxiety

In the first multi-site phase 3 trial of MDMA-assisted therapy (MAT) for PTSD, the primary outcome was change in PTSD symptom severity on the Clinician-Administered PTSD Scale (CAPS-5). MAT showed a large effect size relative to placebo plus therapy^16^. In the same trial, a secondary outcome (Sheehan Disability Scale) showed significantly greater improvements in functional impairment (work, social, and family life) in the MAT arm. A separate qualitative study of MDMA-assisted therapy for anxiety associated with life-threatening illness reported that participants felt ‘reborn into life’ three months after treatment, describing increased vitality, improved quality of life, feeling ‘more resourced,’ and a renewed connection to life^49^. These outcomes were central to patients’ recovery narratives, yet were absent from the primary endpoints.

### Psilocybin for depression

A landmark 2021 randomized trial of psilocybin (25 mg, two sessions) versus escitalopram for moderate-to-severe major depressive disorder illustrates the problem of relying solely on symptom-based primary outcomes^36^. The study’s pre-registered endpoint, the between-group difference in change in QIDS-SR16 depression scores at six weeks, did not significantly separate the treatments. Notably, however, the trial’s other depression measures, the clinician-rated 17-item Hamilton Depression Rating Scale (HAM-D-17) and the MADRS, as well as the self-rated BDI, favored psilocybin, though these between-group comparisons were not adjusted for multiplicity. The primary symptom measure therefore did not significantly separate the treatments, but the other depression scales favored psilocybin—making the overall symptom picture more nuanced than a characterization of ‘equivalence’ would suggest, particularly given that the trial was not designed or powered as a formal equivalence test

This led some commentators to characterize the trial as showing only “equivalence” on its main endpoint. Yet this narrow interpretation overlooked a wide range of secondary and exploratory outcomes on which psilocybin was superior to escitalopram. In the 6-week trial, psilocybin outperformed escitalopram on well-being (Warwick-Edinburgh Mental Wellbeing Scale (WEMWBS)), work and social functioning (Work and Social Adjustment Scale (WSAS)), psychological flourishing, avoidance, anxiety, and other domains of quality of life. Because these were analyzed as secondary/exploratory endpoints (without multiplicity control), they should be interpreted as hypothesis-generating. This highlights why well-being should be prespecified as a primary or co-primary endpoint and trials powered accordingly.

The six-month follow-up study provides a clearer picture of how the two treatments diverge beyond depression symptom scores^13^. Although psilocybin and escitalopram yielded similar long-term reductions in depressive symptom severity (QIDS-SR-16), the psilocybin group showed marked and statistically robust advantages on several secondary measures of well-being and functioning: greater improvements in work and social functioning (WSAS), psychological connectedness (Watts Connectedness Scale (WCS)), and meaning in life (Meaning in Life Questionnaire (MLQ)) across the entire 6-month period. These effects were large and consistent across all follow-up timepoints.

### Well-being as both an endpoint and a pathway

A further argument for measuring well-being concerns timing. In time-limited trials, symptom change may emerge only late, whereas changes in functioning, coping, connectedness, and meaning can appear earlier. These early changes may be more than incidental: in the antidepressant literature, early improvement in positive affect has been found to predict later reduction in depressive symptoms more strongly than early improvement in negative affect, suggesting that positively valenced change may index a resilience-like process rather than merely lag behind symptom relief^50^. Self-transcendent states such as awe, widely regarded as a central feature of the psychedelic experience, illustrate the same possibility: awe engages a set of processes (shifts in neurophysiology, a diminished focus on the self, prosocial relationality, social integration, and a heightened sense of meaning) that have been linked to subsequent improvements in mental health, including depression and anxiety^51,52^. We do not claim the temporal relationship is one-directional. Early symptom relief may equally promote later gains in well-being and psychological flexibility, and the largest acute antidepressant effects in recent psilocybin trials occur within a day of dosing before partly decaying; the direction and durability of these dynamics remain open empirical questions. The practical implication holds regardless of direction: capturing well-being and related change-process variables, and the timing of their movement relative to symptom change, yields information that symptom-only endpoints cannot. This is true whether that information identifies early predictors of response, mediators of recovery, or benefits that outlast symptom change.

## Proposal for a consensus measure

A major barrier to making well-being a primary endpoint is the diversity of views on how to measure well-being, and the proliferation of scales^53^. A comprehensive 2016 review identified 99 generic adult well-being measures spanning widely divergent theoretical models and domains and concluded, *“ …* that there is little consistent agreement on how well-being should be measured, how instruments should be designed or which dimensions should be included”^54^. Similar concerns were raised by the NIH emotional well-being networks^10^. Within this broad and heterogeneous landscape, researchers have gravitated toward a smaller number of prominent but still non-standardized instruments, including the 5-item WHO Well-Being Index (WHO-5), the 8-item Flourishing Scale by Diener et al., the 14-item Warwick-Edinburgh Mental Wellbeing Scale (WEMWBS), the longer Mental Health Continuum (MHC-SF), and the Emotional State Assessment Tool (ESAT). However, even within narrower subdomains, consensus remains elusive. For example, a recent scoping review identified seven distinct flourishing scales in use, varying in their composition of hedonic (happiness, life satisfaction) and eudaimonic (meaning, relationships, accomplishment) domains^55^. This diversity reflects rich academic exploration, but it poses a practical challenge: without standardization, trials will choose different well-being measures, diluting comparability and impact.

We propose to define and validate a brief, multi-domain, and culturally adaptable well-being scale that can serve as a core primary outcome across trials^56^. The outcome would be a proposed “Consensus Well-Being Scale” (CWS)—hypothetically, containing items such as “I feel satisfied with my life overall,” “I have a sense of purpose and meaning,” “I feel connected to other people,” “I am able to cope with life’s challenges,” each rated on a simple Likert agreement scale. The CWS should be scored to yield a single summary of well-being (with subdomains as needed) and importantly should have a known minimally important difference (the smallest change that patients consider meaningful).

First, we propose convening a Delphi consensus process to develop one or more candidate scales. The panel would bring together clinical researchers, psychometric experts, patients, FDA representatives, National Institutes of Health / Patient-Centered Outcomes Research Institute (NIH/PCORI) methodologists, payers, global health metricians and cross cultural psychologists, to agree on: (1) the domains of well-being most broadly important and most predictive of present or future health, and (2) items that reliably capture those domains in diverse populations. The aim is to build on existing validated instruments, merging their strengths into a consensus measure. The value of this process lies not in inventing novel item content but in conferring field-wide and regulatory legitimacy: the resulting instrument, whether an existing scale, a hybrid, or a newly developed measure, would emerge from a transparent, multi-stakeholder process with fresh validation evidence. It could therefore be adopted as a default primary endpoint in a way that no single existing scale, however well validated, currently can.

We emphasize a two-step evidentiary sequence, operationalized as shown in the Supplementary Material: the instrument must first demonstrate sound psychometric properties including reliability, structural and construct validity, and measurement invariance, in the clinical populations in which it will be used, including depression, and not only in general-population samples. Only then can it be used to demonstrate that the well-being construct itself changes within those populations in response to treatment (responsiveness to meaningful change).

This approach has well-established precedents. A core outcome set (COS) is an agreed, minimum set of outcomes that all trials in a field are encouraged to measure. Delphi-based methods are routinely used to build such sets and, in some cases, to recommend specific instruments, for example, the Harmonising Outcome Measures in Eczema (HOME) initiative in dermatology and Outcome Measures in Rheumatology (OMERACT) in rheumatology^57–59^. In mental health, the International Consortium for Health Outcomes Measurement (ICHOM) used a structured modified Delphi to develop a standardized outcome set for depression and anxiety, including selection of patient-reported measures^60^. Our process would similarly extend recent COS work for depression, which began by eliciting outcome domains from thousands of patients, informal caregivers, and clinicians across 52 countries before refining them^5^. Consistent with best practice, we treat the Delphi as the content-validity and stakeholder-legitimacy step and then apply psychometric methods to empirically reduce items, test dimensionality, and evaluate cross-cultural measurement equivalence^61^. See the Supplementary Material for a summary of how a Delphi process, combined with statistical analysis and new survey data, could yield an FDA-recognized consensus well-being measure.Table1.

**Table 1:** Summary of the proposed Delphi-plus-validation roadmap

Phase	Activity	Approx. months
1	Scoping, governance, regulatory consultation	0–6
2	Delphi Round 1 (domain identification)	6–9
3	Delphi Round 2 (item selection, with pruning)	9–12
4	Delphi Round 3 and regulatory checkpoint	12–15
5	Pilot testing and item reduction	15–24
6	Confirmatory validation, invariance testing	24–36
7	Responsiveness and MID derivation	30–42
8	Criterion validation and trial integration	36–54
9	Regulatory documentation and dissemination	48–60+

Total estimated duration is 4–6 years; phases overlap and some can proceed in parallel. The Supplementary Material provides full detail.

To minimize participant burden, such a scale should be brief, ideally 5 to 20 items, consistent with widely-used instruments such as the WHO-5, the Warwick-Edinburgh Mental Wellbeing Scale (WEMWBS), the Mental Health Continuum–Short Form (MHC-SF), and the Flourishing Scale^62^, yet broad enough to capture the core domains of positive mental health^63^. The recurring facets that anchor the candidate item pool (for example, positive affect, meaning and purpose, social connectedness, mastery/accomplishment, and vitality) are detailed in the Supplementary Material; here we note only that they inform, without constraining, the panel’s judgments.

Second, the candidate item pool would undergo psychometric validation that combines expert-driven content validity with empirical item reduction and cross-validation. Dimensionality and item performance would be evaluated (for example, through exploratory and confirmatory latent-variable modeling), poorly performing or redundant items dropped, and the resulting structure validated in independent samples, with tests of measurement equivalence across key subgroups and of responsiveness to change. Several candidate instruments, including the Flourishing Scale, WHO-5, and WEMWBS, already demonstrate strong psychometric properties across multiple languages and contexts. The Supplementary Material specifies this validation sequence in operational detail.

Once defined, the third step would be prospective validation in clinical trials. In parallel, sponsors would engage FDA early to align on the intended context of CWS use and the circumstances under which improvements in positive well-being could support a clinically meaningful claim (e.g., as co-primary with a symptom endpoint or under prespecified non-inferiority ‘guardrails’). The CWS could initially be included as a secondary endpoint to build an evidence base linking it to established outcomes and functioning. Over time, this could support its use as a co-primary (or, in selected contexts, primary) endpoint. Sponsors could also pursue formal qualification of the instrument through FDA’s Clinical Outcome Assessment/Drug Development Tool (COA/DDT) program for a specified context of use—reducing uncertainty about measurement acceptability—while recognizing that any labeling claim would remain contingent on the full trial evidence and prespecified analyses^37^. We propose engaging FDA early and iteratively to align the instrument’s context of use and evidentiary expectations (optionally through the COA/DDT qualification pathway), recognizing that FDA does not endorse specific patient reported outcome (PRO) measures and that qualification/posting is not an endorsement of a tool’s use.

Our proposal is therefore for a *single, brief general well-being scale*, a common core that can be used across mental health trials and mapped to economic and policy frameworks, rather than an ever-expanding menu of incomparable positive endpoints. Condition-specific modules or composites can be layered on only when clearly justified, and should anchor to the core well-being construct.

## Guardrails for regulatory use

Our goal is to enhance, not upend, regulation. We see well-being measures as an adjunct to the ongoing use of formal symptom scales. The two types of measures can be analyzed either as co-primary endpoints, or with hierarchical (“gatekeeping”) testing, in which the well-being endpoint is tested formally only if the symptom endpoint first meets its prespecified success criterion. This preserves protections against approving therapies that worsen the underlying condition while allowing trials to capture direct patient-centered benefit—consistent with FDA’s framing of clinical benefit as improvement in how patients feel and function.

In the uncommon event of discordant results—e.g., the investigational arm shows superior well-being but inferior on the primary symptom endpoint—we recommend a prespecified “discordance rule.” For example, a plausible rule might be, if symptom worsening exceeds (i) a predefined non-inferiority margin or (ii) a minimal clinically important difference (MCID) threshold, the trial should be interpreted as not supporting a disorder-treatment indication, regardless of well-being gains. If symptom differences are statistically unfavorable but remain within the non-inferiority/MCID bounds, then well-being superiority can be considered only alongside (a) corroborating improvements in functioning/global improvement, (b) no excess serious adverse events, and (c) sensitivity analyses addressing expectancy/unblinding (e.g., results robust to adjustment for treatment-guessing and differential dropout/rescue medication use).

We note that blinding is difficult, and often impossible, to achieve fully in psychedelic trials, with correct-guess rates approaching 95% in macrodose studies^64^. However, the relationship between unblinding and therapeutic outcomes may be more complex than is often assumed. The only macrodose trial to formally test the expectancy–outcome association found that, despite substantially higher pre-trial expectations for psilocybin than for escitalopram, expectancy did not predict therapeutic response to psilocybin on any outcome, including both symptom and well-being measures, whereas it did predict escitalopram response, consistent with the broader SSRI literature^65^. Attenuation of between-arm differences after adjusting for expectancy was driven by a nocebo-like effect in the escitalopram arm rather than an inflated effect in the psilocybin arm. These findings are preliminary and require replication with validated instruments and larger samples. Strategies such as low-dose comparators or active placebos (e.g., niacin or methylphenidate) partially mitigate unblinding, and should be complemented by routine measurement of pre-trial expectations so that their contributions can be quantified rather than assumed. Well-being endpoints should be subject to the same expectancy-focused sensitivity analyses applied to symptom endpoints, and trial interpretation should treat unblinding as a shared limitation across all endpoints rather than a distinctive weakness of well-being measures.

## Policy implications

Implementing well-being as a primary endpoint has broad implications for research, regulation, and healthcare delivery:

### Drug and therapy approvals

FDA would take well-being outcomes into consideration for review processes, and regulatory labeling would reflect the blended use of symptomatic and well-being measures. Such an approach could enable claims about patient-experienced benefit without implying approval for use in otherwise healthy populations. This boundary also answers a reasonable concern about coverage: would elevating well-being expand reimbursement to treatments marketed simply for “improving well-being,” and if so, who would be eligible? Our proposal does not create a free-standing well-being indication. Because well-being serves as a co-primary or hierarchically tested endpoint behind prespecified guardrails, approval and coverage remain anchored to a diagnosed condition; the well-being endpoint differentiates the quality and completeness of benefit among treatments for that condition rather than licensing treatment of its absence in otherwise healthy people. Consider two antidepressants equally efficacious on a standard symptom scale such as the MADRS, where one lacks the affect blunting and sexual dysfunction associated with the other. The second would plausibly yield greater well-being, and patients, clinicians, and payers might reasonably prefer and be willing to pay a premium for it. Such differentiation occurs entirely within the depression indication and requires no new eligibility category. Where a therapy improved well-being without improving the underlying disorder, the discordance rules described above would withhold a disorder-treatment indication. Payers and purchasers increasingly seek evidence that treatments improve outcomes patients directly experience, especially functioning and recovery in daily life. A validated, consensus well-being endpoint could support value-based arrangements that reward durable improvements in patient-reported recovery. A substantial employer and labor-economics literature links higher well-being to better work outcomes; longitudinal data show that baseline well-being predicts subsequent productivity and healthcare utilization; and experimental evidence that exogenously increasing happiness can raise task productivity^66^. As a longer-term methodological agenda, such measures could also be evaluated for possible incorporation into economic frameworks. If evidence continues to accumulate that higher well-being predicts lower risk of subsequent mental illness, suicidal behavior, and premature mortality^22,23,25,26,67,68^ as well as lower preventable hospitalizations and healthcare costs^69,70^, payers will have increasingly tangible reasons to favor therapies that deliver durable well-being gains.

### Clinical practice and public health

If trials elevate well-being, clinical practice and treatment guidelines should follow, encouraging clinicians to monitor patient well-being alongside symptom severity, and shifting resources toward interventions that enhance positive mental health rather than merely suppress symptoms. In turn, measurement-based care could incorporate brief well-being questionnaires at routine visits, helping providers aim not just for symptom reduction but for genuinely fulfilling lives. Emphasizing well-being in clinical trials also aligns with public health priorities that increasingly value positive mental health, not just disease reduction. A validated well-being measure could help community and preventive programs demonstrate upstream impacts, reinforcing broader well-being–oriented policy frameworks already adopted in some countries.

### The path forward

Changing primary endpoints requires buy-in from trial sponsors, who may worry about regulatory risk if a novel endpoint is used. This underscores why the consensus-building and validation is essential—to give sponsors confidence that a well-being measure is acceptable to regulators and worth the effort. There is an encouraging precedent: despite many competing instruments, a small set of symptom measures has become *de facto* standards for trials and regulatory submissions (e.g., CAPS-5 for PTSD; HAM-D/MADRS/QIDS-SR for depression). Well-being is currently in a pre-consensus phase, with 99 generic well-being instruments and 196 proposed distinct dimensions^54^. The aim of a Delphi process, therefore, is not to reinvent measurement but to accelerate the same winnowing and convergence that mental health symptom measures have already undergone, yielding one, or a small set, of field-endorsed well-being measures that regulators, payers, and trialists can treat as a default.

## Conclusion

Current psychiatric trial design prioritizes symptom reduction, yet patients consistently report that their treatment goals extend beyond symptom relief to include improved relationships, restored function, and renewed sense of purpose. Validating well-being measures as primary endpoints could further align clinical research with these priorities.

The timing appears favorable. The FDA has established frameworks for patient-reported outcomes, and employers and payers actively seek interventions that demonstrate value beyond traditional clinical metrics. However, the field currently lacks consensus on which well-being instruments possess the psychometric properties needed for primary endpoints, how to establish meaningful change thresholds, and how to balance comprehensiveness with practical trial constraints.

A structured consensus process would systematically address these gaps by evaluating existing instruments, establishing standardized protocols, and defining clinically significant outcomes with demonstrated predictive validity for healthcare utilization, productivity, and long-term health. The objective is not to replace symptom-focused measures but to complement them with an outcome that captures what recovery means to patients.

## Supplementary information

Supplementary_Information

## Data Availability

No datasets were generated or analysed during the current study.
