# Supplementary material for: Well-being as a primary endpoint in clinical trials: a call for consensus
Source: Npj Ment Health Res. 2026 Aug 6;5:39. doi: 10.1038/s44184-026-00234-1 (PMC13462081; doi:10.1038/s44184-026-00234-1)
Supplement: Supplementary file 1 [file 44184_2026_234_MOESM1_ESM.pdf]

Supplementary Information - Delphi Roadmap for Developing a Consensus Well-Being Scale and Validation Plan

This appendix outlines an end-to-end roadmap for developing a brief, culturally adaptable well-being measure that can serve as a primary or co-primary endpoint in behavioral health trials and related studies. The roadmap combines a structured Delphi consensus process with psychometric development and validation methods, including exploratory item reduction, confirmatory modeling, cross-validation, measurement invariance/differential item functioning analyses, and criterion/predictive validation in pragmatic settings. It also specifies how the resulting measure would be integrated into trial designs with appropriate endpoint hierarchies and regulatory safeguards.

The Delphi process may conclude that an existing validated instrument, with or without modification, meets the consensus criteria; the roadmap accommodates this outcome and does not presuppose development of a novel scale.

The roadmap is organized into nine phases with approximate timelines. Total estimated duration from initiation to regulatory-ready documentation is 4–6 years, though phases overlap and some can proceed in parallel. These estimates are approximate only. Actual time will depend on the relative complexity or simplicity of the undertaking as it unfolds. For example, if an existing instrument is deemed adequate, the duration could be substantially reduced.

Phase 1: Scoping, governance, pre-specification, and initial regulatory consultation (Months 0–6)

- Define the intended context(s) of use (e.g., depression trials, PTSD trials, transdiagnostic behavioral health trials), target populations, and administration mode(s) (self-report; electronic/paper; language requirements).
- Establish governance: steering committee, Delphi panel composition targets (clinical, psychometric, patient/participant, payer/HTA, and regulatory-facing expertise), conflict-of-interest handling, and decision rules.
- Define *a priori* measurement goals: brevity (e.g., 5–20 items), recall period, response scale (e.g., 5–7 point Likert), scoring approach options (total score vs. subscales), and thresholds for interpretability (e.g., minimally important difference; responder definition).
- Compile candidate domains and items from existing well-being and positive mental health instruments and from qualitative sources (patient interviews, focus groups, narrative accounts), ensuring cultural and linguistic adaptability. The literature identifies several recurring facets across influential frameworks and widely used instruments—including positive emotion/affect, meaning and purpose, social connectedness, mastery/accomplishment, and vitality—which can inform the candidate materials presented to panelists without constraining their judgments.

- Initiate early regulatory engagement (e.g., pre-submission meeting or informal consultation with FDA's Study Endpoints and Labeling Development division) to align on the intended context of use, evidentiary expectations, and potential applicability of the Clinical Outcome Assessment/Drug Development Tool (COA/DDT) qualification pathway.

Phase 2: Delphi Round 1 — Domain identification and prioritization (Months 6–9)

- Provide panelists with a structured domain list drawn from the candidate compilation (Phase 1), along with an open-text option for nominating additional domains. Ask panelists to rate candidate domains on importance, clarity, distinctiveness, and feasibility for brief measurement.
- Use predefined consensus thresholds (e.g., median rating and interquartile range criteria) to retain, revise, or drop domains.
- Synthesize and feedback results (including minority viewpoints) to panelists to support iterative convergence without suppressing heterogeneity.

Phase 3: Delphi Round 2 — Item selection, dimensionality, and scoring intent (Months 9–12)

- For each retained domain, present candidate items drawn from existing validated instruments and any new items generated in Round 1. Ask panelists to rate each item on content validity, readability, cultural transferability, and redundancy.
- Elicit explicit judgments about whether the construct is best represented as unidimensional (single total score) or multidimensional (subscales with or without a higher-order factor).
- Identify a preliminary scoring intent (e.g., total score plus optional subdomain reporting) and a preliminary interpretation framework (directionality, anchors).
- Assess retained items for potential redundancies while preserving domain coverage and readability. Ensure retained items are suitable for translation and for populations with varying literacy and clinical severity.

Phase 4: Delphi Round 3 — Finalize domain set, structural model, context(s) of use, and regulatory checkpoint (Months 12–15)

- Finalize the core domain set, the hypothesized structural model (e.g., bifactor, second-order, correlated factors), and the final intended context(s) of use. The Delphi may conclude that an existing instrument, a modified version of an existing instrument, or a novel measure best serves as the consensus endpoint; all three outcomes should be treated as legitimate.

- Confirm the administration specifications (recall period, response scale, mode, language plan) and finalize pre-specified analytic decisions (e.g., candidate Item Response Theory (IRT) model class; invariance groups; planned external anchors).
- Document consensus outputs transparently (panel composition, response rates, consensus criteria, and rationale for retained/dropped domains and items).
- Conduct a regulatory checkpoint: share Delphi outputs with FDA (e.g., through the COA/DDT qualification pathway or an informal pre-submission interaction) to confirm alignment between the emerging measure and evidentiary expectations before committing to large-scale data collection.

Phase 5: Pilot testing and exploratory factor analysis (Months 12–24)

- Conduct cognitive interviews to test comprehension, response processes, and item interpretability; revise wording and instructions as needed.
- Field a pilot survey in a heterogeneous sample adequate for stable exploratory estimation (size depending on items and model; typically several hundred).
- Perform exploratory factor analysis and/or exploratory IRT screening to identify poorly performing items (e.g., low discrimination, extreme difficulty/thresholds, local dependence) and to reduce redundancy.
- Assess preliminary acceptability and burden (completion time, missingness, floor/ceiling effects).

Phase 6: Confirmatory validation, cross-validation, and measurement invariance (Months 24–36)

- Collect a sufficiently large and representative dataset for confirmatory evaluation (e.g., $N > 500$, larger if multiple groups or complex models are planned).
- Fit confirmatory factor analytic models and/or IRT models consistent with the Phase 3 structural hypothesis; evaluate global and local fit, item parameter stability, and score reliability across the trait range.
- Use internal cross-validation to test stability of the chosen model and scoring approach.
- Evaluate reliability: internal consistency (as appropriate for the model) and test–retest reliability over a clinically sensible interval in a stable subgroup.
- Establish convergent and discriminant validity against pre-specified comparator constructs (symptoms, functioning, life satisfaction, quality of life), using a pre-registered portfolio of expected associations.

- Test measurement invariance and/or differential item functioning (DIF) across key groups (e.g., age, sex/gender, race/ethnicity, language, diagnosis, site, and administration mode) concurrently with confirmatory analyses, provided the sample is sufficiently diverse. Where non-invariance/DIF is detected, revise items, consider group-specific parameters, or adopt scoring adjustments consistent with the intended context(s) of use.
- Implement a translation and linguistic validation workflow (forward/back translation, harmonization, and cognitive debriefing) for planned language versions.

Phase 7: Responsiveness, interpretability, and thresholds for meaningful change (Months 30–42)

- Evaluate responsiveness to change in interventional and observational settings; estimate effect sizes and standardized response means. This phase requires interventional data and should be coordinated with early deployments in Phase 8.
- Derive minimally important difference (MID) and/or responder definitions using anchor-based methods (e.g., global rating of change, functional milestones) complemented by distribution-based evidence.
- Assess floor/ceiling effects and identify score ranges where interpretability is strongest; specify how to report total and subdomain scores in publications.

Phase 8: Criterion and predictive validation in trials, including endpoint integration (Months 36–54)

- Deploy the consensus measure in clinical trials and pragmatic cohorts to evaluate associations with outcomes that matter to patients and systems (e.g., functioning, return to work/school, relapse/recurrence, suicidality, healthcare use).
- Test incremental validity: whether well-being scores predict these outcomes beyond symptom scales and baseline covariates.
- Evaluate longitudinal measurement properties (growth modeling, sensitivity to sustained change, and temporal ordering relative to symptom change).
- Specify and pilot trial designs in which the consensus measure serves as a co-primary or hierarchically tested key secondary endpoint alongside a symptom measure. This includes:
    - Pre-specified multiplicity-adjustment strategies (e.g., hierarchical/gatekeeping procedures, alpha splitting) for co-primary or key secondary configurations.
    - Sample size planning that powers the trial to detect meaningful change on the well-being endpoint, not only on the symptom endpoint.

- Pre-specified discordance rules that define how to interpret cases in which the symptom and well-being endpoints yield conflicting results—for example, requiring that symptom worsening not exceed a predefined non-inferiority margin or clinically important difference threshold, and that well-being superiority be considered only alongside corroborating evidence (functioning, global improvement, no excess serious adverse events, and sensitivity analyses addressing expectancy/unblinding).

- These trial-integration specifications operationalize the guardrails described in the main manuscript and should be documented in statistical analysis plans prior to unblinding.

Phase 9: Fit-for-purpose documentation, regulatory submission, and dissemination (Months 48–60+)

- Prepare documentation suitable for regulatory-facing use (instrument description, content validity evidence, scoring manual, interpretation guidance, and evidence supporting the stated context(s) of use).
- If appropriate, pursue formal qualification through FDA's COA/DDT program for the specified context of use, thus reducing uncertainty about measurement acceptability, while recognizing that qualification is not an endorsement and that any labeling claim would remain contingent on the full trial evidence and prespecified analyses.
- Develop additional materials for researchers and implementers (short form, translations, scoring code, user manual) and define a governance plan for future revisions and version control.

Summary timeline

Phase	Activity	Approximate months
1	Scoping, governance, regulatory consultation	0–6
2	Delphi Round 1	6–9
3	Delphi Round 2 (with pruning as needed)	9–12
4	Delphi Round 3 and regulatory checkpoint	12–15
5	Pilot testing and item reduction	15–24
6	Confirmatory validation, invariance testing	24–36
7	Responsiveness and MID derivation	30–42

Phase	Activity	Approximate months
8	Criterion validation and trial integration	36–54
9	Regulatory documentation and dissemination	48–60+

Phases 6–8 overlap substantially, as confirmatory data collection, responsiveness testing, and early trial deployment

Appendix B - Regulatory context for positive well-being endpoints

FDA: Increasing receptivity to patient-centered outcomes. Over the past decade, the U.S. Food and Drug Administration (FDA) has expanded its commitment to patient-focused drug development [1,2]. Through the 21st Century Cures Act, multiple Prescription Drug User Fee Act (PDUFA) agreements, and the four-part Patient-Focused Drug Development (PFDD) guidance series, the FDA has strengthened expectations that sponsors measure outcomes reflecting how patients *feel and function* in daily life. These initiatives formalize what FDA leaders have emphasized: patients are "experts in living with their disease," and their priorities should help determine endpoints. In psychiatry, the commonly used symptom measures are themselves PROs, but they overwhelmingly capture negative states. What is missing is a validated pathway for sponsors to use positive mental health outcomes as primary indicators of benefit. The barrier is not regulatory resistance but evidentiary standards: measures of well-being must demonstrate reliability, validity, and a clear link to direct clinical benefit. If those criteria are met, positive well-being outcomes could meaningfully shape both regulatory decision-making and labeling, since FDA labeling depends on endpoints deemed to reflect clinically meaningful benefit. Elevating well-being measures into this evidentiary tier would allow sponsors to support claims that a therapy improves patients' overall health status, not only reduces symptoms

Centers for Medicare & Medicaid Services **(CMS) & Medicare: Scaling patient-reported outcomes in payment and performance programs.**

CMS is increasingly operationalizing patient-reported outcome measures (PROMs) at national scale and tying them to accountability and payment—an important precedent for any future use of patient-centered endpoints, including positive mental well-being. A clear example is the Total Hip/Total Knee Arthroplasty Patient-Reported Outcome Performance Measure (THA/TKA PRO-PM), which requires hospitals to collect pre- and post-operative patient-reported pain and function data and submit them for public reporting and payment programs [3]. After an initial voluntary period, CMS moves to mandatory reporting, with payment consequences for hospitals that do not report sufficient data [3]. Similarly, Medicare Advantage plans administer the Health Outcomes Survey (HOS), which captures patient-reported physical and mental health functioning and is used in plan quality measurements [4]. These CMS measures largely focus on pain, function, and health status rather than positively-valanced well-being, but they demonstrate the infrastructure and policy willingness to use patient-reported outcomes as consequential measures in federal programs

Private Payers & Employers: Demand for functioning and flourishing.

Commercial insurers and self-insured employers are also increasingly focused on outcomes that matter to the people they cover – their members and employees. In employer healthcare purchasing, there is a growing emphasis on "presenteeism" (being fully engaged and productive at work) in addition to traditional absenteeism. Effective mental health interventions can yield significant returns in productivity: for instance, analyses have found that for every $1 invested in treating depression and anxiety, there is about a $4 return in improved work ability and reduced lost productivity [5]. Large employers have taken note of these figures. As a result, employer-sponsored plans and coalitions are pushing for whole-person health outcomes from vendors and

providers [6]. They are asking not just "Does this reduce PTSD symptoms?" but "Does this help our employees lead healthier, more fulfilling lives and perform better?" Insurance payers are likewise exploring "value-based insurance design" where patient-reported health status improvements could trigger better reimbursement terms for providers or lower copays for patients [7,8].

References

1. U.S. Food and Drug Administration. Patient-Reported Outcome Measures: Use in Medical Product Development to Support Labeling Claims: Guidance for Industry. Accessed August 20, 2025. https://www.fda.gov/regulatory-information/search-fda-guidance-documents/patient-reported-outcome-measures-use-medical-product-development-support-labeling-claims
2. U.S. Food and Drug Administration. Focus Area: Patient-Reported Outcomes and other Clinical Outcome Assessments. Accessed August 19, 2025. https://www.fda.gov/science-research/focus-areas-regulatory-science-report/focus-area-patient-reported-outcomes-and-other-clinical-outcome-assessments#:~:text=Strengthening%20FDA%E2%80%99s%20ability%20to%20use,making
3. Plate JF, Deen JT, Deans CF, Pour AE, Yates AJ, Sterling RS. Implementation of the New Medicare-Mandated Patient-Reported Outcomes After Joint Arthroplasty Performance Measure. *The Journal of Arthroplasty*. 2024;39(5):1136–1139. doi:10.1016/j.arth.2024.01.038
4. Centers for Medicare & Medicaid Services. Health Outcomes Survey (HOS). Accessed August 19, 2025. https://www.cms.gov/data-research/research/health-outcomes-survey
5. Chisholm D, Sweeny K, Sheehan P, et al. Scaling-up treatment of depression and anxiety: a global return on investment analysis. *Lancet Psychiatry*. May 2016;3(5):415–24. doi:10.1016/S2215-0366(16)30024-4
6. Business Group on Health. Taking Action on Health Care Costs: A Business Group on Health Viewpoint. Accessed August 20, 2025.
7. Squitieri L, Bozic KJ, Pusic AL. The Role of Patient-Reported Outcome Measures in Value-Based Payment Reform. *Value Health*. Jun 2017;20(6):834–836. doi:10.1016/j.jval.2017.02.003
8. Fendrick AM, Chernew ME, Levi GW. Value-based insurance design: embracing value over cost alone. *Am J Manag Care*. Dec 2009;15(10 Suppl):S277–83.
